# Acute Encephalitis Caused by Intrafamilial Transmission of Enterovirus 71 in Adult

**DOI:** 10.3201/eid1405.071121

**Published:** 2008-05

**Authors:** Tsuyoshi Hamaguchi, Hironori Fujisawa, Kenji Sakai, Soichi Okino, Naoko Kurosaki, Yorihiro Nishimura, Hiroyuki Shimizu, Masahito Yamada

**Affiliations:** * Kanazawa University Graduate School of Medical Science, Kanazawa, Japan; † Ishikawa Prefecture Central Hospital, Kanazawa, Japan; ‡ Fujii Neurosurgical Hospital, Kanazawa, Japan; § Ishikawa Prefectural Institute of Public Health and Environmental Science, Kanazawa, Japan; ¶ National Institute of Infectious Diseases, Tokyo, Japan

**Keywords:** entervirus 71, encephalitis, intrafamilial transmission, adult, dispatch

## Abstract

Enterovirus 71 (EV71) is a common cause of hand, foot, and mouth disease and sometimes causes severe neurologic complications, mainly in children. We report a case of adult-onset encephalitis caused by intrafamilial transmission of a subgenogroup C4 strain of EV71. This case elucidates the risk for EV71 encephalitis even in adults.

In children, enterovirus 71 (EV71) is a common cause of hand, foot, and mouth disease (HFMD), and most patients recover within 4–6 days. However, severe neurologic complications, such as acute encephalitis and poliolike paralysis, develop in some patients with EV71 infection. In the largest and most severe EV71-associated HFMD outbreak occurring in Taiwan in 1998, 405 children had severe neurologic complications, pulmonary edema, or both; 78 children died (*1*). In adults, transmission of EV71 within households is common, but EV71 infection is commonly limited to mild illness, and neurologic complications are uncommon in adults (*2*–*4*). We report a case of acute EV71 encephalitis in a mother and cases of HFMD in her 3 sons due to intrafamilial transmission of EV71.

## The Case

**Figure 1:**
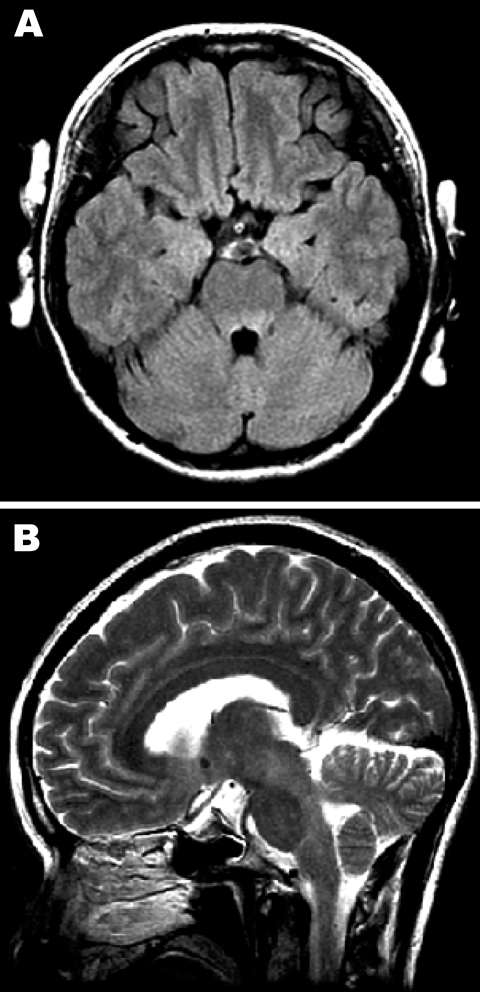
Magnetic resonance images of the brain. A) Hyperintense lesions in the tegmentum of the pons in the axial section of the fluid-attenuated inversion recovery image. B) In the sagittal section of the T2-weighted image, hyperintense lesions are present in the tegmentum of the midbrain, pons, and medulla oblongata.

In November 2006, a 37-year-old woman without serious past illness sought treatment at our hospital with hand tremor, unsteadiness, and a 2-day history of headache (day 1). Examination showed high fever (39.3°C), neck stiffness, intentional tremor of bilateral upper extremities, and truncal ataxia. Brain magnetic resonance images (MRI) and results of laboratory blood tests were normal. A cerebrospinal fluid (CSF) tap showed 305 leukocytes/mm^3^ (82.5% polymorphonuclear leukocytes and 17.5% lymphocytes) and total protein concentration of 62 mg/dL with normal glucose levels. Empirical therapy with acyclovir and cefotaxime was initiated. On day 4, the patient reported diplopia and slurred speech. Ocular movements were not obviously restricted, and the extremities showed ataxia without weakness. She could not sit on the bed without support because of severe unsteadiness. Deep tendon reflexes were absent, and the patient had no pathologic reflexes. Brain MRI showed hyperintense lesions in the tegmentum of the medulla oblongata, pons, and midbrain in T2-weighted and fluid attenuated inversion recovery images (Figure 1). No abnormalities of the cervical spinal cord were detected on MRI. Results of nerve conduction studies were within normal ranges except for the absence of an F-wave in the median and ulnar nerves. Methylprednisolone (1 g/day) was administered for 3 days. From day 5 and forward, the patient gradually improved. A CSF tap on day 15 showed 14 leukocytes/mm^3^ (100% lymphocytes) and a total protein concentration of 50 mg/dL. On day 22, MRI showed that the brain had normalized. Three months after the onset of disease, she had completely recovered.

During the illness, CSF was negative for bacteria and viruses. Enterovirus-specific RNA was detected from a stool sample on day 16 by a seminested reverse transcription–PCR (RT-PCR) with consensus-degenerative primers from Nix et al. (*5*); the virus was identified as EV71 by sequence analysis of the partial VP1 region (*5*). Serum neutralizing antibody titer against EV71 increased, from 8 on day 1 to 128 on day 15. There was no increase in serum antibodies against other viruses, including herpes simplex virus, cytomegalovirus, varicella-zoster virus, Epstein-Barr virus, rubella virus, and mumps virus by enzyme immunoassay, and against Japanese encephalitis virus by hemagglutination-inhibition test. Results for antinuclear antibody and antiganglioside antibodies were also negative.

Three days before the patient sought treatment, her 1-year-old son was affected with HFMD. This disease also developed in her other 2 sons, 5 and 7 years of age, on day 2. Her 3 children recovered within several days without any neurologic complications. Enterovirus-specific RNA was also detected in the stool samples from the 3 children by seminested RT-PCR (*5*), and all 3 viruses were identified as EV71 by sequence analysis. In addition, EV71 was isolated in Vero cells from stool samples from 2 of the 3 sons with HFMD. Stool and other clinical samples from the mother were all negative for virus isolation on Vero and RD cells.

**Figure 2:**
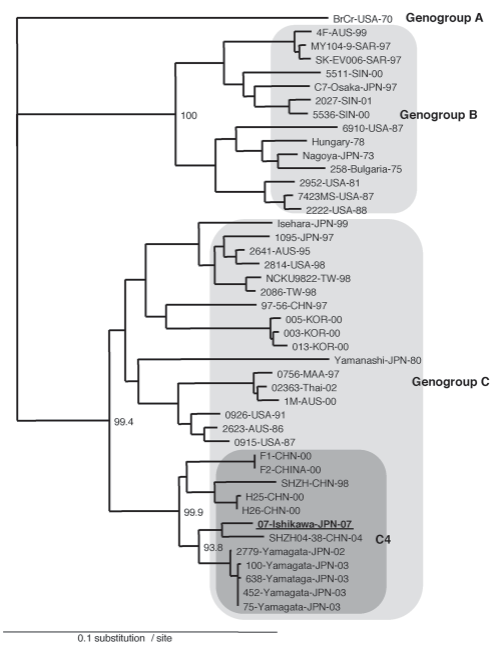
Phylogenetic analysis of EV71 based on the entire VP1 sequences. The tree was prepared by the neighbor-joining method by using the EV71 strains in the world as described previously (*6*) and newly identified subgenogroup C4 strains (*7**,**8*) were also included in the analysis.

The partial VP1 sequences (150 bp) of the PCR products directly amplified from stool samples of all 4 cases were 100% identical. The entire VP1 sequences (891 bp) of the EV71 isolates from 2 of her sons with HFMD (07-Ishikawa and 08-Ishikawa) were also 100% identical. Phylogenetically, by using VP1-based genetic classification, the isolates were classified as subgenogroup C4 (Figure 2) (*6*). The subgenogroup C4 of EV71 has recently been identified in Japan and might have emerged in the surrounding countries, mainland People’s Republic of China and Taiwan (*6*–*9*). The 07-Ishikawa strain shows a close genetic relationship to recent subgenogroup C4 strains in mainland China (97.4% nt identity to the SHZH04–38 strain) and those in Japan (97.0% nt identity to the 2779-Yamagata strain, Figure 2) (*7*,*9*).

## Conclusions

Several EV71 outbreaks have been documented throughout the world, and clinical manifestations of EV71 infections can be diverse, including HFMD, herpangina, central nervous system (CNS) complications, and pulmonary edema. Recently, EV71-associated HFMD outbreaks with severe CNS complications have frequently been reported, especially in the Asian-Pacific region (*2*,*3*). In children, the CNS complications associated with EV71 manifest clinically in various ways, such as aseptic meningitis, acute flaccid paralysis, and rhombencephalitis; rhombencephalitis is one of the most common severe neurologic symptoms (*2*).

We diagnosed the mother’s illness as EV71 encephalitis because the clinical features were similar to those of EV71 rhombencephalitis in children (*2*,*10*), although there has not previously been a detailed case report of adult-onset EV71 encephalitis. EV71 was not identified in the mother’s CSF sample by virus isolation or direct molecular detection by RT-PCR, but EV71 was identified in her stool sample. We could not exclude the possibility of para- or post-infectious encephalitis during the initial stage of her illness. However, rhombencephalitis subsequently developed in this patient, which is common in children with EV71 encephalitis but is far less common from other infections and para- or post-infectious encephalitis. Clinical symptoms, MRI, and CSF findings of her illness were similar to those reported in children with EV71 encephalitis.

Several previous studies have demonstrated a rather low virus isolation rate in CNS specimens compared with that in other clinical samples, such as throat swab, rectal swab, and stool samples from EV71-associated cases with HFMD, encephalitis, or both (*2*,*3*,*11*). Along with the identification of EV71, the increase in serum neutralizing antibody titer against EV71 supports the diagnosis of acute EV71 infection. In addition, the lack of abnormalities of the spinal cord on MRI, the absence of an F-wave on nerve conduction study (a possible sign of radiculopathy), and the absence of deep tendon reflexes without weakness support the possibility of radiculitis as a complication. In a previous report, some patients with rhombencephalitis showed hyporeflexia or areflexia, but nerve conduction study findings were not reported (*2*).

Genetic analysis among 4 different EV71 isolates from the patients indicated probable intrafamilial transmission of EV71. In a recent study, EV71 transmission rate to household contacts was 52%, and the transmission rate from children to parents was 41% (*4*). Twenty-one percent of EV71-infected children experienced serious complications, including CNS or cardiopulmonary failure. By contrast, 53% of adults were asymptomatic, and all symptomatic adults recovered completely from uncomplicated illnesses (*4*). Considerable attention has been paid to EV71 infection in children because young age was considered the major risk factor associated with severe CNS complications, such as encephalitis resulting in severe neurologic sequelae and deaths (*2*–*4*,*12*). Thus, less attention has been paid to the adult-onset EV71 encephalitis. Our patient showed a good prognosis; however, a 19-year-old man died from EV71 encephalitis in Singapore (*3*). More careful disease surveillance, even for adults, will be needed during EV71-associated HFMD outbreaks.

## References

[1] Ho M, Chen ER, Hsu KH, Twu SJ, Chen KT, Tsai SF, An epidemic of enterovirus 71 infection in Taiwan. N Engl J Med. 1999;341:929–35. 10.1056/NEJM199909233411301 10498487

[2] Huang CC, Liu CC, Chang YC, Chen CY, Wang ST, Yeh TF. Neurologic complications in children with enterovirus 71 infection. N Engl J Med. 1999;341:936–42. 10.1056/NEJM199909233411302 10498488

[3] Chan KP, Goh KT, Chong CY, Teo ES, Lau G, Ling AE. Epidemic hand, foot, and mouth disease caused by human enterovirus 71, Singapore. Emerg Infect Dis. 2003;9:78–85. 12533285 10.3201/eid1301.020112 PMC2873753

[4] Chang LY, Tsao KC, Hsia SH, Shih SR, Huang CG, Chan WK, Transmission and clinical features of enterovirus 71 infections in household contacts in Taiwan. JAMA. 2004;291:222–7. 10.1001/jama.291.2.222 14722149

[5] Nix WA, Oberste MS, Pallansch MA. Sensitive, seminested PCR amplification of VP1 sequences for direct identification of all enterovirus serotypes from original clinical specimens. J Clin Microbiol. 2006;44:2698–704. 10.1128/JCM.00542-06 16891480 PMC1594621

[6] Shimizu H, Utama A, Onnimala N, Li C, Li-Bi Z, Yu-Jie M, Molecular epidemiology of enterovirus 71 infection in the Western Pacific Region. Pediatr Int. 2004;46:231–5. 10.1046/j.1442-200x.2004.01868.x 15056257

[7] Mizuta K, Abiko C, Murata T, Matsuzaki Y, Itagaki T, Sanjoh K, Frequent inportation of enterovirus 71 from surrounding countries into the local community of Yamagata, Japan, between 1998 and 2003. J Clin Microbiol. 2005;43:6171–5. 10.1128/JCM.43.12.6171-6175.2005 16333123 PMC1317214

[8] Lin KH, Hwang KP, Ke GM, Wang CF, Ke LY, Hsu YT, Evolution of EV71 genogroup in Taiwan from 1998 to 2005: an emerging of subgenogroup C4 of EV71. J Med Virol. 2006;78:254–62. 10.1002/jmv.20534 16372302

[9] Li L, He Y, Yang H, Zhu J, Xu X, Dong J, Genetic characteristics of human enterovirus 71 and coxsackievirus A16 circulating from 1999 to 2004 in Shenzhen, People’s Republic of China. J Clin Microbiol. 2005;43:3835–9. 10.1128/JCM.43.8.3835-3839.2005 16081920 PMC1233905

[10] Shen WC, Chiu HH, Chow KC, Tsai CH. MR imaging findings of enteroviral encephalomyelitis: an outbreak in Taiwan. AJNR Am J Neuroradiol. 1999;20:1889–95. 10588115 PMC7657785

[11] Chang LY, Lin TY, Huang YC, Tsao KC, Shin SR, Kuo ML, Comparison of enterovirus 71 and coxsackievirus A16 clinical illnesses during the Taiwan enterovirus epidemic, 1998. Pediatr Infect Dis J. 1999;18:1092–6. 10.1097/00006454-199912000-00013 10608631

[12] Chang LY, Huang LM, Gau SS, Wu YY, Hsia SH, Fan TY, Neurodevelopment and cognition in children after enterovirus 71 infection. N Engl J Med. 2007;356:1226–34. 10.1056/NEJMoa065954 17377160

